# Neurotoxicity: A Rare Side Effect of Programmed Cell Death 1 (PD-1) Inhibitors

**DOI:** 10.7759/cureus.22584

**Published:** 2022-02-24

**Authors:** Syed Ehsanullah, Syed Hasan, Faran S Polani, Syeda zarmeena Rashid, Syed Ijlal Ahmed

**Affiliations:** 1 Medicine, Washington University School of Medicine, St. Louis, USA; 2 Oncology, Washington University School of Medicine, St. Louis, USA; 3 Internal Medicine, Washington University School of Medicine, St. Louis, USA; 4 Internal Medicine, Dow University of Health Sciences, Dow International Medical College, Karachi, PAK; 5 Neurology, Saint Louis University School of Medicine, St. Louis, USA

**Keywords:** non-small cell lung cancer (nsclc), lung cancer, anti-pd-1, immunotherapy adverse effect, immuno-checkpoint inhibitor

## Abstract

Immunotherapy is a biological therapy that helps the body’s immune system to fight against cancer cells. The Food and Drug Administration (FDA) approved the first immune checkpoint inhibitor in 2011. Since 2011, many immune checkpoint inhibitors have been approved. Programmed cell death 1 (PD-1) inhibitors are now commonly used in multiple malignancies due to their remarkable response. Thus, immune-related adverse events are now coming into the limelight due to the increasing use of PD-1 inhibitors. Here, we present a case of a 54-year-old female with non-small cell lung cancers (NSCLC) treated with pembrolizumab and later presented with severe neurotoxicity.

## Introduction

The Food and Drug Administration (FDA) approved programmed cell death 1 (PD-1) inhibitors such as nivolumab and pembrolizumab for the treatment of stage IV non-small cell lung cancers (NSCLC) in 2014 [1]. Pembrolizumab is a humanized monoclonal antibody that works against the negative immunoregulatory human cell surface receptor PD-1, whereas nivolumab is a human immunoglobulin G4 monoclonal antibody that is also directed against PD-1 [1]. Common immune-related adverse events (irAEs) receiving PD-1 inhibitors include skin, colon, endocrine organs, liver, and lungs [1,2]. The adverse effects are thought to be related to the development of autoimmunity [3]. Neurological toxicities are rare with PD-1 inhibitor use, and there is not much data available in the published literature. This case highlights and educates clinicians about the rare neurological adverse effects of PD-1 inhibitors.

## Case presentation

**Figure 1:**
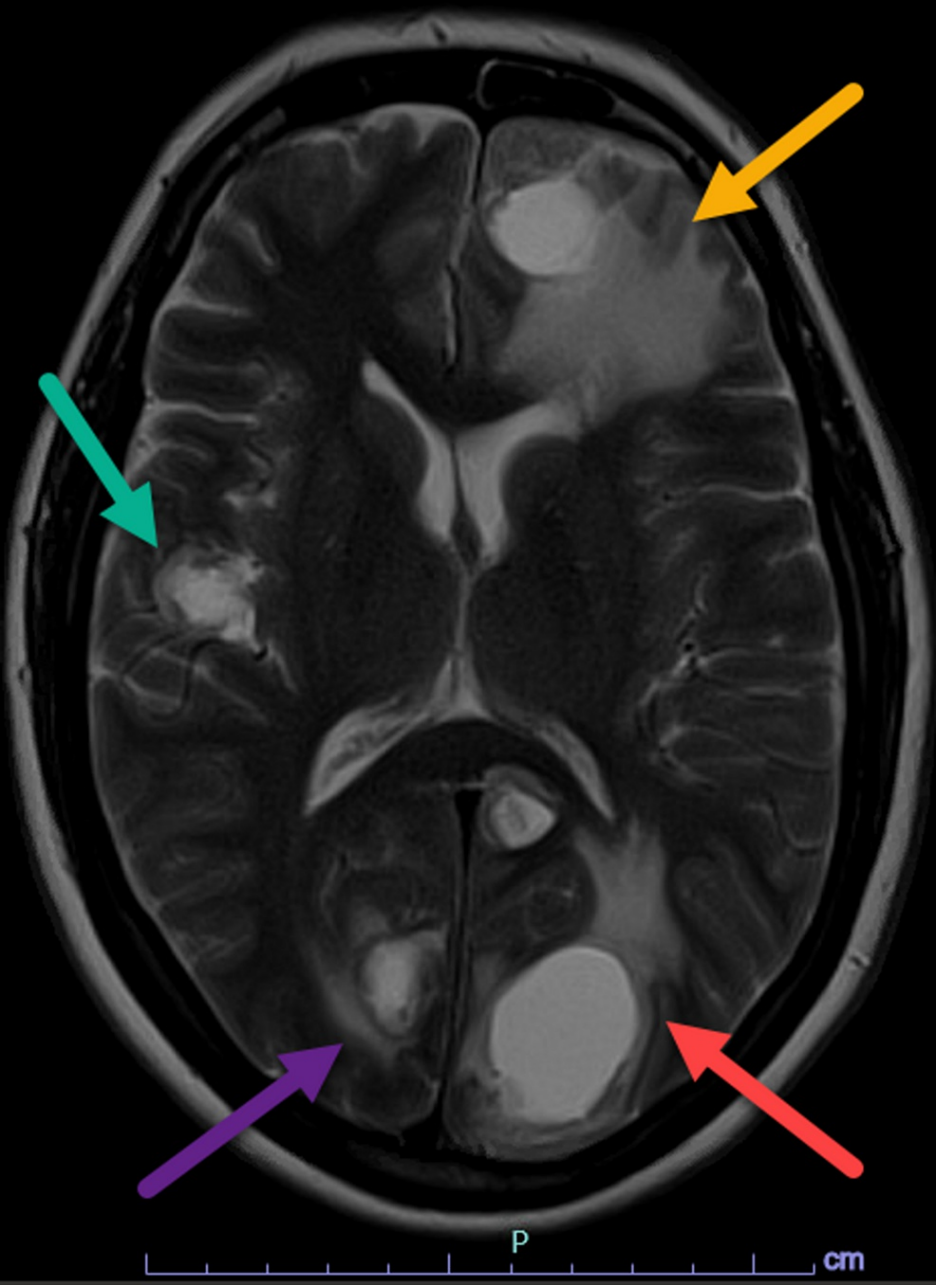
Pre-treatment axial T2 MRI demonstrating multifocal T2 hyperintense lesions involving left occipital (red arrow), right occipital (purple arrow), left frontal (yellow arrow), and right parietal lobe (green arrow) with associated perilesional edema and mass effect

A 54-year-old female with a past medical history significant for stage IV NSCLC with brain metastasis was presented to the emergency department with the complaint of confusion and altered mental status. Her vital signs, blood chemistry, thyroid-stimulating hormone, vitamin B12, and folate levels were within the normal limits in the emergency department. No source of infection was identified. A computed tomography (CT) scan in the emergency department demonstrated post-treatment changes without any acute intracranial abnormality. For her NSCLC metastatic disease, she was initially treated with 10 fractions of whole-brain radiation followed by four cycles of carboplatin, paclitaxel, and pembrolizumab. Her pre-treatment magnetic resonance imaging (MRI) scan demonstrated hyperintense lesions involving the occipital, left frontal, and right parietal lobe with perilesional edema and mass effect (Figure 1). Currently, she is on pembrolizumab maintenance therapy.

**Figure 2:**
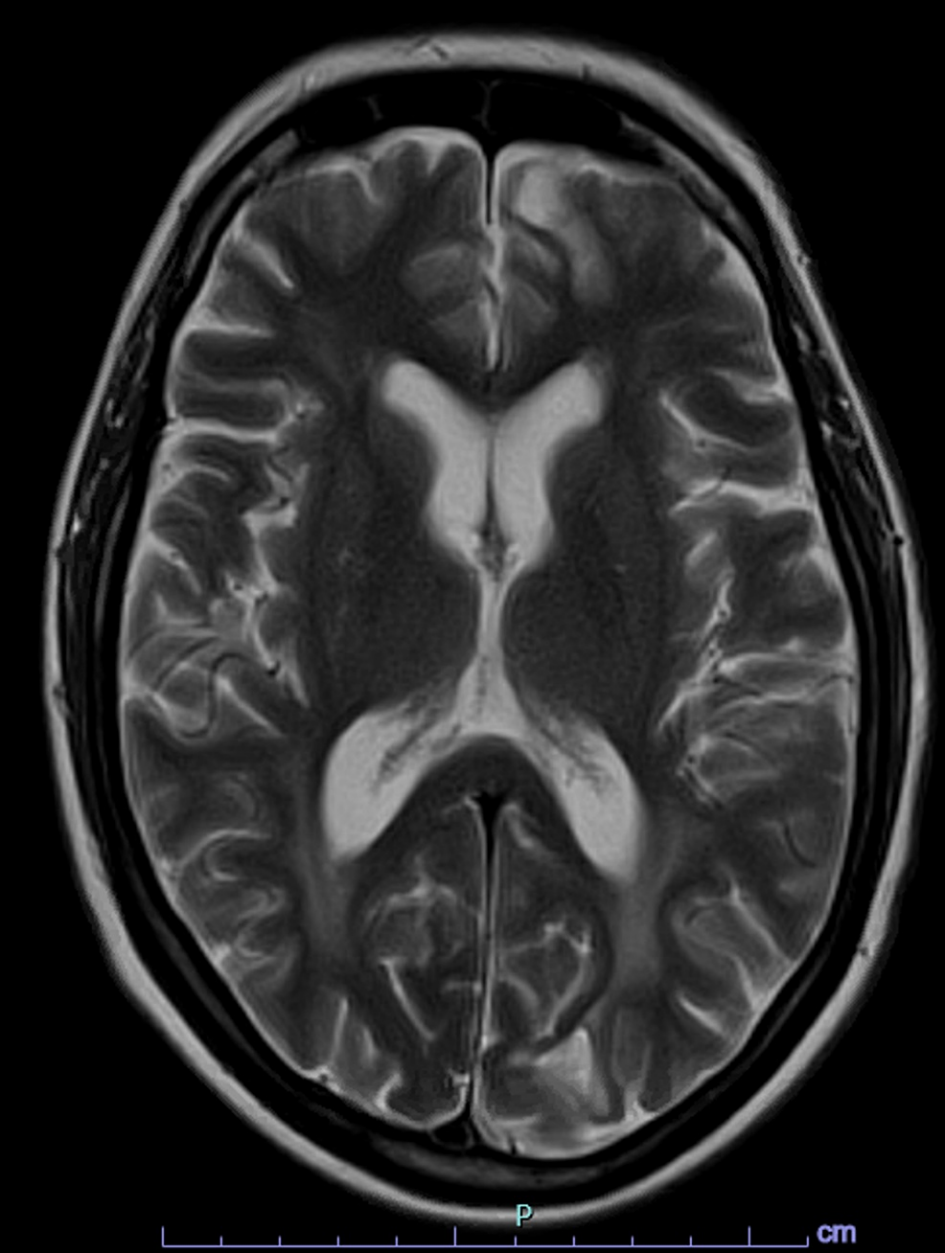
Post-treatment axial T2 MRI image demonstrating the resolution of previously noted T2 hyperintense lesions with mild residual edema in bilateral occipital and left frontal lobes

During this hospitalization, a brain MRI revealed intracranial metastasis with interval decrease in size and a stable decrease in the surrounding vasogenic edema and nonspecific findings of pachymeningeal enhancement with no definite nodular focus and no additional areas of intracranial progression (Figure 2). A positron emission tomography (PET) scan showed interval resolution of previously identified left and infra hilar lesions, suggestive of treatment response. Cerebrospinal fluid studies were negative for infectious and paraneoplastic etiologies. Continuous electroencephalography (cEEG) monitoring showed two brief self-limiting episodes of focal seizures. The patient was started on high-dose intravenous methylprednisolone. She showed remarkable improvement and was later discharged from the hospital after full recovery with slow steroid taper over six weeks. The inpatient oncology team suggested no further immunotherapy due to severe neurological toxicity.

## Discussion

The use of immunotherapy in treating patients with cancer is becoming more common because of its promising results. The cytotoxic T-lymphocyte-associated antigen 4 (CTLA4) and the PD-1 receptor as well as programmed death-ligand 1 (PD-L1) are the two most effective immune checkpoint inhibitor targets. These immune checkpoints negatively control T-cell-mediated immunity and have critical roles in immune homeostasis and in preventing autoimmunity [4,5]. Inhibition of physiologic checkpoints results in cytotoxic T-cell activation, which leads to tumor death [4]. T-cell upregulation leads to disruption of immune tolerance, ultimately causing autoimmune syndromes in various host tissues. Common irAEs are thyroiditis, hypophysitis, hepatitis, pneumonitis, colitis, and a local or generalized body rash. Others are very infrequent such as neurological disorders and myocarditis, which may be very serious or even lethal [1,2]. Neurological toxicity secondary to immunotherapy use is seen in 4.2% of cancer patients [6].

There are only seven cases reported on neurological irAEs with the use of anti-PD-1 in patients with NSCLC [1]. Among those seven cases, neurological symptoms were cerebellar ataxia [6], headache [6], myasthenia gravis [7-9], encephalitis [10], and encephalopathy [3]. Myasthenia gravis is emerging neurotoxicity with the use of PD-1 inhibitors, with 30.4% of related mortality [11]. Our patient also had neurotoxicity, altered mental status, focal seizures, and confusion. Extensive imagining and blood and cerebrospinal fluid analysis ruled out other possible causes of altered mental status such as infectious, paraneoplastic, and metabolic processes. Patients should also be tested for other reversible causes like vitamin B12 or folate deficiencies, thyroid-stimulating hormone (TSH) impairment, and human immunodeficiency virus (HIV) infection.

The irAEs are divided into three grades depending on the severity: mild symptoms (grade 1), moderate symptoms (grade 2), and severe symptoms or life-threatening adverse reactions (grade 3). Generally, management is based on the severity of the symptoms. Grade 1 irAEs are managed by holding the PD-1 agent and supportive care. Grade 2 irAEs are managed by prednisone 0.5-1 mg/kg or hospital admission. Grade 3 is managed by therapy with intravenous methylprednisolone 2-4 mg/kg in addition to duloxetine, pregabalin, or gabapentin for the pain and discontinuation of immunotherapy until recovery at least to grade 1 [12]. Our patient had a grade 3 adverse event and received a high dose of intravenous methylprednisolone that was slowly tapered over the course of six weeks. She showed remarkable improvement and was later discharged from the hospital after a full recovery. The oncologist suggested no further immunotherapy due to severe neurological toxicity.

## Conclusions

Immune checkpoint inhibitors are becoming essential in the therapeutic strategies of cancer treatment. Patients can present with a wide variety of symptoms. We present this case to highlight and educate about rare neurological adverse effects of PD-1 inhibitors. Prompt detection and management of irAEs are salient in minimizing the drug-related adverse effects and helping in the clinical recovery of the patient.
